# Supplementary material for: Failure to replicate the diabetes alleviating effect of a maternal gluten-free diet in non-obese diabetic mice
Source: PLoS One. 2023 Sep 8;18(9):e0289258. doi: 10.1371/journal.pone.0289258 (PMC10490983; doi:10.1371/journal.pone.0289258)
Supplement: Attachment — Submitted filename: Response to reviewers.docx [file pone.0289258.s002.docx]

**Letter of response to the comments from the academic editor and reviewers**

Ms. Ref. No.: PONE-D-23-01826

We wish to thank the editor and reviewers for the valuable and relevant comments and questions. We have made revisions of the manuscript (marked in red) in accordance with the reviewers’ suggestions. This letter outlines in detail the changes made in the manuscript, referring point-by-point to the questions/criticisms of the reviewers and the editors.

Additional Editor Comments:

This paper was fairly judged by two judges. Unfortunately, the results were judged to be insufficient for publication in this paper.

Although we respect the editor’s opinion, we are puzzled about the reasons to reject and would like to challenge the decision with the following arguments:

1. Both reviewer 1 and 2 agree that the manuscript is technically sound and that the quality of the statistics, presentation of data, and language is high/sufficient.
2. Reviewer 2 applause us for submitting our manuscript reporting a negative finding. Moreover, reviewer 2 is overall positive and has suggestions for improving the manuscript that we have now implemented in the manuscript (se comments below). In our opinion, the changes have improved the manuscript.
3. In our opinion reviewer 1 has unfortunately misunderstood a few central points. First, the purpose of our study was not to try and repeat the previous finding of a gluten-free diet having an alleviative effect for type 1 diabetes. The purpose was to further dissect the mechanisms behind. Moreover, to adequately show that a gluten-free diet alleviates diabetes it is in our opinion important to show that the effect is stable; hence, the effect should not be vendor specific etc. We strongly disagree with reviewer 1 that the study did not provide any useful information, as this statement seems purely to be based on the fact that we could not replicate previous findings. However, the study was conducted in exactly the same way and with the same high quality as our previous studies that found an effect of the gluten-free diet.
4. PLOS ONE encourage researcher to share their negative and null results: https://everyone.plos.org/2020/04/06/filling-in-the-scientific-record-the-importance-of-negative-and-null-results/
5. A gluten-free diet has show some anti-diabetogenic effects in both humans (interventions studies and epidemiological studies) as well as in mouse models. However, some studies do not find this effect, as we openly report in our manuscript. We know that other groups have had problems showing that a gluten-free diet reduces the autoimmune diabetes incidence in NOD mice but they have not published their work. Our collaborators studying the anti-diabetic effect of a gluten-free diet has encouraged us to publish our negative results. In our opinion, not publishing our result would basically be bad research practice and can potentially lead to the believe that a gluten-free diet is anti-diabetogenic, when it may not be or may be less anti-diabetogenic than we previously believed. Hence, not publishing these results could ultimately have consequences for the type 1 diabetes patients, which is why it is so important to get our results out for the type 1 diabetes community to decide.

Comments from the Reviewer:

Reviewer #1: For this current study, the authors tired to repeat their previous published results. However, the authors used a different animal vendor, a different GF food, and a different assay for gluten detection. They could not figure out the cause of the discrepancies in the diabetes alleviate effect of the GF diet intervention between the current and previous studies. Basically, this current study did not provide any useful information. Therefore, I would suggest to reject this manuscript.

Thank you for the comments. The purpose of our study was not to try and repeat the previous finding of a gluten-free diet having an alleviative effect for type 1 diabetes. The purpose was rather to further dissect the mechanisms behind. Moreover, to adequately show that a gluten-free diet alleviates diabetes it is in our opinion important to show that the effect is stable; hence, the effect should not be vendor specific etc. We respectfully disagree with you that the study did not provide any useful information, as this statement seems to be based on the fact that we could not replicate previous findings. However, the study was conducted in exactly the same way, with the same diet recipe, and with the same high quality as our previous studies that found an effect of the gluten-free diet. Moreover, a gluten-free diet has shown some anti-diabetogenic effects in both humans (interventions studies and epidemiological studies) as well as in mouse models. However, some studies do not find this effect, as we openly report in our manuscript. We know that other groups have had problems showing that a gluten-free diet reduces the autoimmune diabetes incidence in NOD mice but they have not published their work. Our collaborators studying the anti-diabetic effect of a gluten-free diet has encouraged us to publish our negative results. In our opinion, not publishing our result would basically be bad research practice and can potentially lead to the believe that a gluten-free diet is anti-diabetogenic, when it may not be or may be less anti-diabetogenic than we previously believed. Hence, not publishing these results could ultimately have consequences for the type 1 diabetes patients, which is why it is so important to get our results out for the type 1 diabetes community to decide.

Reviewer #2: This is a negative study showing that a gluten free (GF) diet during the pregnancy of NOD mice had no effect on the rate of diabetes development in 1st generation offspring. There were also no differences in glucose and insulin tolerance, insulitis scores, insulin autoantibody titres in the offspring of GF and standard mouse diet (STD) treated pregnant NOD mice.

It is important to report negative studies, so I applaud the authors for submitting this m/s for publication.

The NOD mice of this study were from the Jackson lab rather than from Taconic. The authors almost dismiss this as the possible reason for the negative result here. The mice of the two different sources are quite different genetically and this may well be the reason. Agree, we have added more about this possible reasons in the conclusion of the discussion.

I have a few comments for improving the manuscript.
1) The title is difficult to follow. Suggest something like: Failure to replicate effects of a maternal gluten free diet in non-obese diabetic mice to reduce rates of offspring type 1 diabetes development. Agree, corrected.
2) Line 59 - should be 'a multifactorial disease'. Agree, corrected.
3) Line 106 - "of 0.01 mL 0.00075 U insulin (Actrapid®, Novo Nordisk, Bagsværd, Denmark) per g body weight does not make sense" Should the '0.00075 U insulin be a concentration (units/violume)? Agree, we have corrected to: 0.00075 U insulin/g body weight
4) Through the results section there are formatting errrors in the PDF " (Error! Reference source not found.a). " These need to be corrected in a new version. Agree, corrected.
5) In results section Lines 121-123 "a significantly higher distribution of insulitis score 1 (few mononuclear cells in an islet) was observed in GF mice compared to STD mice (236%, p=0.0032, U=14, 95.67% confidence interval (3.4%;7.7%))" doesn't make sense to me. Is there a clearer way to present these results. It is also very unclear if there is any real biological significance of this result. Could insulitis Score 0 and 1 be combined? We would like to stay with the current way of showing our results, as it is the usual way. We would rather not combine score 0 and 1, as these are different degrees of insulitis.
6) In Figure 2, it would be better to present actual glucose levels rather than % from baseline as this is more informative and can allow comparison to other studies. Were mice that were already diagnosed with diabetes excluded from these tests?? We would prefer to stay with %, as our lab normally reports the data that way. This allows us/the readers to compare our current and previous results easily. Only normoglycemic mice were used for analysis, as now specified in the method section.

Yours sincerely,

Martin Haupt-Jørgensen
