# Supplementary material for: Failure to replicate the diabetes alleviating effect of a maternal gluten-free diet in non-obese diabetic mice
Source: PLoS One. 2023 Sep 8;18(9):e0289258. doi: 10.1371/journal.pone.0289258 (PMC10490983; doi:10.1371/journal.pone.0289258)
Supplement: Attachment — Submitted filename: Response to reviwers.docx [file pone.0289258.s003.docx]

**Letter of response to the comments from the academic editor and reviewers**

Ms. Ref. No.: PONE-D-23-01826R1

We wish to thank the editor and reviewers for the valuable and relevant comments and questions. We have made revisions of the manuscript (marked in red) in accordance with the reviewers’ suggestions. This letter outlines in detail the changes made in the manuscript, referring point-by-point to the questions/criticisms of the reviewers and the editors.

Specifically, the authors should address the following points:

(1) The suthors state that " The current study was initiated to further explore the mechanism behind the reported diabetes alleviating effect of a GF diet in utero". However, the used methodology are quite similar to that used in previous research. Therfore, the authors should explain how they would "further explore the mechanisms" using the same methods. Did the authors plan for more advanced or additional studies but they didn't conduct them given the "negative" results? If this is the case, authors should explain.

Additional experiments were originally planned to explore the diabetes alleviative effect of the GF diet intervention, including analysis of microbial composition of cecal content, histological examination of different intestinal segments, IHC for inflammation markers in intestinal tissue and verification of microbial/inflammatory markers in plasma. These were, however, not performed due to the negative findings, which we write in the abstract, line 31-33: “In conclusion, this study could not replicate the previously observed diabetes alleviative effects of a maternal gluten-free diet in NOD mouse offspring and could therefore not further elucidate potential mechanisms.” and in the discussion, line 195-197: “Hence, the mechanism behind the diabetes-alleviating effect of a GF diet in utero, which we intended to explore in this study, is dependent also on other factors.”. Moreover, we have added the following sentence in the discussion, line 198-202: “Initially, this will require a large-scale animal study using NOD mice from different vendors, testing the possible diabetes-alleviative effects of a strictly GF diet initiated at different time points, including analyzing both the intestinal microbiota composition and histology at different prediabetic ages and the diet gliadin levels with a reliable analysis kit.”.

(2) In the discussion, the authors provided some good possible explanations for why they coud not replicate the results of their previous studies. However, the whole manuscript gives the impression that the the positive findings of the authors' previous studies are the "correct", although it is possible that the "positive" findings in the previous studies could be due to type 1 error, and the "negative" findings in this study could be due to type 2 error.

We have focused on laying forward our results in an unbiased way throughout the manuscript. However, we agree that the discussion could be more moderate and have thus now included/modified the following sentence in line 195-197: “Hence, the mechanism behind the diabetes-alleviating effect of a GF diet in utero, which we intended to explore in this study, is dependent also on other factors.”

(3) While the the authors provided some possible explanations for the inconsistent findings between this and the previous studies, they should elaborate on the implications of such findings on both the clinical and research levels with future researrch directions, particulary as the authors conclude that "the possible diabetes-alleviative effect of a GF diet should be further explored". For example, what precautions/points the researchers should be aware of when they perform similar studies in the future? should we do such study using mice from different vendors? Should we compare different gliaden detection kits? Should future studies assess microbiota composition? etc...

We agree on this. Hence, we have implemented the following sentence, line 198-202: Initially, this will require a large-scale animal study using NOD mice from different vendors, testing the possible diabetes-alleviative effects of a strictly GF diet initiated at different time points, including analyzing both the intestinal microbiota composition and histology at different prediabetic ages and the diet gliadin levels with a reliable analysis kit.

Moreover, for the question if we should compare different gliadin detection kits, also see line 163-165. Furthermore, see the following article comparing different gliadin detection analysis kits: ”Comparative study of commercially available gluten ELISA kits using an incurred reference material” Z. Bugyi et al 2013 Quality assurance and safety of crops and foods.

(4) Authors have to acknowldge some study limitations. For example, if the main aim of the study was to "further explore the mechanism behind the reported diabetes alleviating effect of a GF diet in utero", they should have strictly followed the same methods of the previous study, including mice from same vendors, although it could be an important finding that the vendor change could possibly affect the findings!. Second, it would be helpful to to do histopathoological studies in the intestine (like the previous studies). Third, assessment of microbiota might have provided useful information.

Thank you for the comment. We can confirm that the main aim was to further explore the mechanisms behind the GF diets diabetes alleviating effect. However, we strongly believed to get the same effect with NOD mice from a different vendor and it was moreover not possible at that point to get a sufficient number of mice from the original vendor that we bought the NOD mice from in our previous studies. These are the reasons why we got the mice from a different vendor. Also, to really trust the diabetes alleviating effect behind the GF diet, the effect should show stable results, including in NOD mice from different vendors, which was not the case, unfortunately. Besides using another vendor and another gliadin detection kit, the procedure used in our different GF intervention studies was perfectly the same.

To address the raised concern from the reviewer, we have implemented the following sentence, line 198-202: Initially, this will require a large-scale animal study using NOD mice from different vendors, testing the possible diabetes-alleviative effects of a strictly GF diet initiated at different time points, including analyzing both the intestinal microbiota composition and histology at different prediabetic ages and the diet gliadin levels with a reliable analysis kit.

(5) (Error! Reference source not found) still appears several times in the results section.

Thank you. It could not be seen in the proof. We believe that it has been corrected now.

6. Review Comments to the Author

Please use the space provided to explain your answers to the questions above. You may also include additional comments for the author, including concerns about dual publication, research ethics, or publication ethics. (Please upload your review as an attachment if it exceeds 20,000 characters)

Reviewer #3: The authors present a very interesting manuscript that is very well written. I believe it is very important to publish this study as the authors show that the findings regarding alleviating diabetes with a gluten-free diet are not yet stable. Thus this study may have a large impact for patients that would be considering a gluten-free diet as an intervention. As these negative results may have great implications and it is in accordance with PLOS ONE recommendations to publish negative results, I recommend this manuscript for publication.

Thank you for the comment.

Reviewer #4: Monsted et al report the failure of reproducing earlier data obtained in NOD mice that have been fed a gluten-free diet in utero. The authors repeated experiments that have been published earlier in a very similar way with the goal to get a more mechanistic insight of how protection from T1D was achieved. Unfortunately, they have not been able to detect any differences between the experimental groups.
As an additional referee I share the opinion of the other two reviewers that the data are experimentally sound and the conclusions made are supported by the provided data. I also share the opinion of reviewer #2 that “negative” data should be published.
However, I also agree with the editor’s decision. Since the work demonstrated that data of an earlier publication of the same research group cannot be confirmed, the only sensible way to handle this dilemma would be to write a short retraction article to the initial published paper, send it to “Diabetes Metab Res Rev” and in fact retract the publication. Thereby the intention of the authors is irrelevant. Fact is that the present manuscript proves that the previous work was invalid and should therefore be retracted. The authors must admit that anything else would be unfair to the scientific community and the peer-review system since the authors would end up having two additional publications on their record that consist of very questionable data. However, once retracted, I suggest that the authors would publish the entire data set as “negative and null results”. This would also be in agreement with the author’s response #3 to reviewer #1, since the robustness of the study (i.e. food and mouse vendor) could be addressed properly.

Thank you for the comment. We do not believe that any methodological mistakes have been made in neither the current nor the previous published studies. Hence, we cannot see the idea in retracting the published articles. We believe that the scientifically correct way of handling the discrepant studies is to publish them, as the reality probably is that a gluten-free diet is not always diabetes alleviative i.e. less stable than previously believed. This is, however, important information that should not be held back. This opinion is moreover based on the human studies, both intervention studies and epidemiological studies that show inconsistent results.

Reviewer #5: The paper describes a failure to replicate the previously shown diabetes-alleviating effects of GF diet on NOD mice offspring while given in utero.
I agree with the authors and reviewer 2 that despite this being a negative study, the methodology is sound and the results are of importance. Failure to replicate results in dietary intervention studies increases current knowledge by highlighting the complexity through which diet can affect the outcomes. The authors discuss possible mechanisms which could have led to the current (negative) results and make effort to deliver explanations. The connection of GF diet and gut microbiota differences between vendors seems like a plausible explanation for the failure to replicate results. However, I think the future reader would appreciate if the authors were able to provide data or to explain (as a limitation) why it was not possible to examine gut microbiota composition of the mice from the current experiment (Jackson Laboratories) and compare them to the results of mice gut microbiota composition from previous successful experiments (Taconic). The possible observed differences might pinpoint the microbial taxa playing a role in relaying the diabetes alleviating effect of GF diet.

Thank you for the relevant comment.

Other groups have found that a gluten-free diet alleviates type 1 diabetes in NOD mice: “A maternal gluten-free diet reduces inflammation and diabetes incidence in the offspring of NOD mice Hansen” CHF et. al. 2014. Hence, it seems that a gluten-free diet has some diabetes alleviate effect, but other factors such as vendor and microbiota affect the outcome.

We agree that it would have been good to have done fecal microbiota analysis in our current and previous studies. However, in the previous studies the fecal microbiota composition was not analyzed, which was also the case in the current one. Hence, a comparison would not have been possible. Instead, in line 198-202, we now suggest that a new thorough study is initiated taking these limitations into considerations: “Initially, this will require a large-scale animal study using NOD mice from different vendors, testing the possible diabetes-alleviative effects of a strictly GF diet initiated at different time points, including analyzing both the intestinal microbiota composition and histology at different prediabetic ages and the diet gliadin levels with a reliable analysis kit.”

Reviewer #6: Even though this research produces negative data and contradicts previous results, I think this article deserves to be published with discussions in the article.

Thank you for the comment.

Yours sincerely,

Martin Haupt-Jørgensen
